# A Rare Congenital Cause of Epilepsy

**DOI:** 10.7759/cureus.11204

**Published:** 2020-10-27

**Authors:** Neethu Gopal, Ayushi Jain, Sukhwinder Johnny S Sandhu, Alok A Bhatt, Erik H Middlebrooks

**Affiliations:** 1 Neurology, Mayo Clinic, Jacksonville, USA; 2 Radiology, Mayo Clinic, Jacksonville, USA; 3 Neuroradiology, Mayo Clinic, Jacksonville, USA

**Keywords:** epilepsy, brain malformation, calvarial malformation

## Abstract

Enlarged parietal foramina (PFM) are congenital calvarial defects characterized by bilateral parietal bone defects (>5 mm), occurring on each side of the sagittal suture along its posterior aspect. While often lacking underlying intracranial malformations, there has been increasing recognition of coexisting brain malformations in certain subtypes. We present a case of a 12-year-old girl presenting with new-onset grand mal seizure with developmental delay and a known family history of epilepsy. Brain MRI revealed large, bilateral parietal bone defects with underlying cortical malformation (polymicrogyria and ulegyria) and vascular abnormalities (persistent falcine sinus), related to PFM. This case report describes the genetic basis for recognized subtypes of PFM and the rare association of brain malformations associated with PFM due to mutations in the ALX4 homeobox gene.

## Introduction

While congenital calvarial disorders are rare developmental anomalies, enlarged parietal foramina (PFM) represent an elusive subtype, with a prevalence of approximately one in 25,000 [1]. These are variable degrees of defective intramembranous ossification of the parietal bone, located close to the intersection of the sagittal and lambdoid sutures. The associated cortical and vascular malformations with enlarged PFM may predispose to epilepsy [1]. We report the imaging features of PFM in a 12-year-old girl, presenting with a new-onset grand mal seizure.

## Case presentation

**Figure 1:**
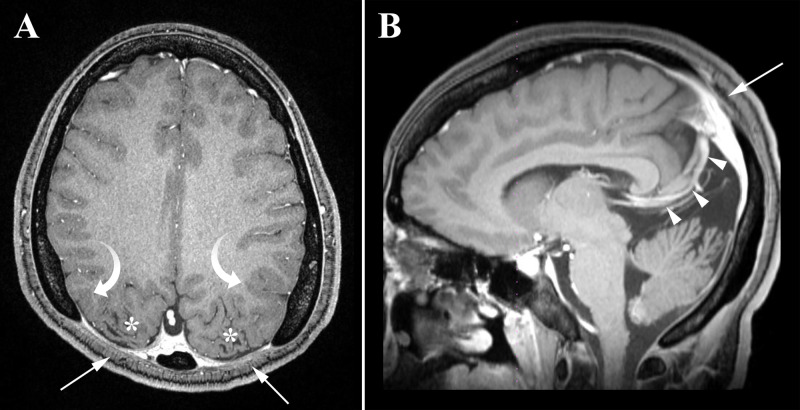
Axial (A) and sagittal (B) post-contrast T1-weighted MRI images show large parietal bone defects (arrows) with underlying cortical malformations including ulegyria (asterisks) and polymicrogyria (curved arrows). A persistent falcine sinus (arrowheads) drains the deep venous system to the superior sagittal sinus and the straight sinus is absent. The falcine sinus is a normal fetal vein that ordinarily involutes after birth. Additionally, there is a large posterior fossa with a high insertion of the tentorium cerebri.

A 12-year-old girl presented to the emergency department with new-onset grand mal seizure. The mother reports that she has had episodes in the past with staring or inattentiveness, but never convulsions. There was a family history of epilepsy and “brain problems.” On exam, she was developmentally delayed, but the neurological exam was otherwise normal. MRI of the brain was performed revealing large, bilateral parietal bone defects with an underlying cortical malformation (polymicrogyria and ulegyria), large posterior fossa with a high insertion of the tentorium cerebri, and vascular abnormalities (persistent falcine sinus) (Figure 1).

## Discussion

The findings present in this case are characteristic of PFM, a rare disorder resulting in variable degrees of defective intramembranous ossification of the parietal bones. In PFM, bilateral parietal bone defects occur on each side of the sagittal suture along its posterior aspect. PFM must be distinguished from the small persistent PFM (<5 mm) that transmit normal anastomotic vessels, which are generally considered benign, incidental findings. PFM has recently been linked to mutations in two separate genes. PFM can be classified by heterozygous mutations of the MSX2 (PFM-1) or ALX4 (PFM-2) homeobox genes [2]. While these mutations may have similar phenotypic manifestations, they are thought to represent distinct clinical syndromes.

MSX2 mutations in type 1 PFM are generally associated with skull deformities, most commonly PFM. Meanwhile, ALX4 mutations seem to represent a spectrum of disorders dependent upon the size and location of the mutation. While some ALX4 mutations may result only in PFM, larger deletions result in Potocki-Shaffer syndrome (PSS) [2]. The required features of PSS are PFM and multiple exostoses, but also may manifest other findings, including brain and facial malformations and severe developmental delay. While PSS represents one extreme of ALX4 mutations, intermediate phenotypes are also reported and commonly associated with co-existing brain and cerebrovascular abnormalities that may lead to epilepsy [3]. Previously reported abnormalities include polymicrogyria, poorly developed straight sinus and persistent median prosencephalic vein, large posterior fossa with high insertion of the tentorium cerebri, and periatrial white matter signal abnormalities.

In this patient, an ALX4 mutation results in a constellation of findings-PFM, ulegyria and polymicrogyria, developmental delay, and a persistent falcine sinus. The falcine sinus is a normal fetal vein connecting the superior sagittal sinus and straight sinus or vein of Galen, which typically involutes after birth. Persistence of the falcine sinus is most commonly an isolated and incidental finding; however, it can less commonly be associated with other abnormalities, such as a vein of Galen aneurysmal malformation or type 2 PFM.

## Conclusions

PFM is a rare, yet important cause of epilepsy. Since PFM is related to mutations in MSX2 or ALX4 gene, the detection of enlarged PFM may have significant consequences for the patient including coexistent malformations. Techniques such as preimplantation genetic diagnosis and MRI to exclude other malformations should be considered as they could reduce potential morbidity for patients with PFM.
